# Long-Read Sequencing Reveals Genetic Adaptation of *Bartonella* Adhesin A Among Different *Bartonella henselae* Isolates

**DOI:** 10.3389/fmicb.2022.838267

**Published:** 2022-02-07

**Authors:** Arno Thibau, Katharina Hipp, Diana J. Vaca, Sounak Chowdhury, Johan Malmström, Athanasios Saragliadis, Wibke Ballhorn, Dirk Linke, Volkhard A. J. Kempf

**Affiliations:** ^1^Institute for Medical Microbiology and Infection Control, University Hospital, Goethe University, Frankfurt am Main, Germany; ^2^Electron Microscopy Facility, Max Planck Institute for Developmental Biology, Tübingen, Germany; ^3^Division of Infection Medicine, Department of Clinical Sciences, Lund University, Lund, Sweden; ^4^Section for Genetics and Evolutionary Biology, Department of Biosciences, University of Oslo, Oslo, Norway

**Keywords:** trimeric autotransporters adhesin, genetic variability, PacBio SMRT sequencing, host matrix-pathogen interaction, *Bartonella* adhesin A

## Abstract

*Bartonella henselae* is the causative agent of cat scratch disease and other clinical entities such as endocarditis and bacillary angiomatosis. The life cycle of this pathogen, with alternating host conditions, drives evolutionary and host-specific adaptations. Human, feline, and laboratory adapted *B. henselae* isolates often display genomic and phenotypic differences that are related to the expression of outer membrane proteins, for example the *Bartonella* adhesin A (BadA). This modularly-structured trimeric autotransporter adhesin is a major virulence factor of *B. henselae* and is crucial for the initial binding to the host via the extracellular matrix proteins fibronectin and collagen. By using next-generation long-read sequencing we demonstrate a conserved genome among eight *B. henselae* isolates and identify a variable genomic *badA* island with a diversified and highly repetitive *badA* gene flanked by *badA* pseudogenes. Two of the eight tested *B. henselae* strains lack BadA expression because of frameshift mutations. We suggest that active recombination mechanisms, possibly via phase variation (i.e., slipped-strand mispairing and site-specific recombination) within the repetitive *badA* island facilitate reshuffling of homologous domain arrays. The resulting variations among the different BadA proteins might contribute to host immune evasion and enhance long-term and efficient colonisation in the differing host environments. Considering the role of BadA as a key virulence factor, it remains important to check consistently and regularly for BadA surface expression during experimental infection procedures.

## Introduction

*Bartonella henselae* is a slow-growing bacterium causing cat scratch disease, a self-limiting zoonotic disease characterised by localised lymphadenopathy or “culture-negative” endocarditis. Infection of immunocompromised patients might result in vasculoproliferative disorders, e.g., bacillary angiomatosis (Relman et al., 1990; Lamps and Scott, 2004). Cats serve as the major reservoir host for *B. henselae* and their infection mostly manifests as asymptomatic bacteraemia. Transmission among cats is predominantly mediated by the cat flea, *Ctenocephalides felis* (Chomel et al., 1996).

The genus *Bartonella* currently consists of more than 40 identified species (Breitschwerdt, 2017; Okaro et al., 2017) and includes both zoonotic and human pathogens with a wide array of mammals as reservoir hosts (Buffet et al., 2013; Kešnerová et al., 2016). *Bartonella* species are haematophagous-arthropod-borne, facultative intracellular α-proteobacteria, and are characterised by their “stealthy” course of infection where host-specific adaptation is essential for their survival (Engel et al., 2011; Harms and Dehio, 2012). As such, long-lasting infections of reservoir hosts are commonly asymptomatic, while incidental host infections often show a clinically apparent course of infection. Homologous recombination (e.g., phase variation) and horizontal gene transfer (HGT) mediate the origination of different substrains within one species, and simultaneously maintain genome integrity (Kyme et al., 2003; Segers et al., 2017; Wagner and Dehio, 2019). Reductive genome evolution is commonly observed within the genus and is concordant with the overall intracellular lifestyle and vector-borne transmission (Ettema and Andersson, 2009).

Consequently, *B. henselae* isolates demonstrate variable genomic and phenotypic differences, presumably driven by adaptation to varying host conditions and caused by frequent recombination events (Lindroos et al., 2006). So far, two *B. henselae* genotypes were proposed based on the 16rRNA gene sequence and are represented by either strain ATCC49882*^T^* Houston-I (genotype I) (Regnery et al., 1992) or strain Marseille (genotype II) (Drancourt et al., 1996). Differing correlations of these genotypes regarding their infection strategy of feline and human endothelial cell lines have been described (Berrich et al., 2011; Chang et al., 2011; Huwyler et al., 2017). Phenotypic differences are among others related to the expression of *Bartonella* adhesin A (BadA) and the VirB/D4 type IV secretion system (Kyme et al., 2003; Lu et al., 2013).

*Bartonella henselae* is characterised by its enormous surface-expressed adhesin BadA which is important for efficient adherence to extracellular matrix (ECM) proteins (e.g., fibronectin and collagen) and host cells, and for angiogenic reprogramming (Riess et al., 2004; Müller et al., 2011; Kaiser et al., 2012). BadA is a trimeric autotransporter adhesin (TAA) (synonyms: non-fimbrial adhesin, oligomeric coiled-coil adhesin, type Vc secretion system) and follows the common TAA-architecture consisting of an N-terminal head, a repetitive and long neck/stalk region, and a more conserved C-terminal membrane anchor (Hoiczyk, 2000; Szczesny and Lupas, 2008; Leo et al., 2012). The long neck/stalk region is composed of several domains which are defined by a distinctive neck sequence functioning as a connector from bulkier β-strands to slimmer α-helixes (Bassler et al., 2015). The modular and repetitive composition of BadA (and other TAAs) suggests the frequent occurrence of recombination, likely as an evolutionary adaptive process in the light of alternating host conditions. Differences in the repetitive neck/stalk region leading to variations in the size of *badA* among several *B. henselae* strains were previously demonstrated (Riess et al., 2007). However, it was not possible to correctly sequence this genomic region because of its highly repetitive nature. Compared to short-read sequencing technologies, long-read sequencing facilitates to differentiate close variants and to cover highly repetitive stretches without major assembly problems (Tørresen et al., 2019).

In this study we report on long-read sequenced whole genomes of eight *B. henselae* isolates, discuss their genomic organisation with a special emphasis on the highly variable *badA* coding region (i.e., *badA* island), and check for BadA expression and functional binding to ECM proteins. These data suggest an adaptive evolution of the *badA* island and confirm the importance of BadA as a virulence factor.

## Materials and Methods

### Bacterial Strains and Culture Conditions

All *B. henselae* strains used in this study are listed in Table 1. Bacteria were grown in *Bartonella* liquid (BALI) medium (Riess et al., 2008) supplemented with 10% sterile fetal calf serum (FCS) for three days in a humidified atmosphere at 37°C and 5% CO_2_ while gently shaking (120 RPM). Alternatively, *B. henselae* strains were cultured on Columbia blood agar (CBA) plates with 5% sheep blood (Becton-Dickinson) for either 4 days to obtain fully grown plates, or for 14 days to obtain single colonies, both in a humidified atmosphere at 37°C and 5% CO_2_. Both growth conditions ensured surface expression of BadA (proven by immunofluorescence; data not shown). Competent *Escherichia coli* DH5α (NEB), used for cloning and plasmid amplification, were grown overnight (o/n) at 37°C either in shaking (180 RPM) Luria/Miller (LB) broth or on LB agar plates (Carl Roth).

**TABLE 1:** Overview of *B. henselae* strains used in this study*^a^*.

*B. henselae* strain	Alternative designation(s)	BadA expression	Complete genome sequence	Specifications	Source
Marseille	URLLY-8	Yes	This study	Isolate from a patient diagnosed with cat scratch disease (Marseille, France)	Drancourt et al., 1996
Marseille BadA-transposon mutant	ΔBadA-T	No	–	*B. henselae* Marseille with a TN < KAN-2 > transposon integrated in *badA*	Riess et al., 2003; Riess et al., 2004
Marseille BadA-deletion mutant	ΔBadA-D	No	–	*B. henselae* Marseille with *badA* deleted via homologous recombination	This study
ATCC49882*^T^* var-1	RSE247; CHDE101; Houston-I variant-1	No	This study and NZ_CP020742.1	Spontaneous laboratory streptomycin-resistant variant of *B. henselae* ATCC49882*^T^* Houston-I (isolate from a febrile patient infected with human immunodeficiency virus, Houston, United States)	Regnery et al., 1992; Schmid et al., 2004
ATCC49882*^T^* var-2	Houston-I variant-2	Yes	This study	Laboratory isolate (1996); variant of ATCC49882*^T^* Houston-I	Riess et al., 2007; Lu et al., 2013
Berlin-I	–	No	This study	Isolate from a skin biopsy specimen from a patient diagnosed with bacillary angiomatosis (Berlin, Germany)	Arvand et al., 1998
G-5436	Houston-I; Zürich	Yes	This study	Human isolate, Centers for Disease Control and Prevention (Atlanta, United States); Possible derivative of *B. henselae* ATCC49882*^T^* Houston-I	Regnery et al., 1992; Zbinden et al., 1995; Zbinden et al., 1997
88-64 Oklahoma	–	Yes	This study	Blood isolate from a patient diagnosed with HIV (Oklahoma City, United States)	Welch et al., 1992
FR96/BK38	Type I	Yes	This study	Blood isolate from domestic cat (Freiburg, Germany)	Sander et al., 1997
FR96/BK3	Type II	Yes	This study	Blood isolate from domestic cat (Freiburg, Germany)	Sander et al., 1997

*^a^Passage number of all strains in Frankfurt am Main was <10. Exact passage number before arriving is unknown.*

As selection marker, kanamycin (KAN; MP Biomedicals) was used at a final concentration of 30 μg/ml (*B. henselae* Marseille ΔBadA-T) and 50 μg/ml (*E. coli* DH5α). Bacteria were collected by centrifugation at 5,000 × *g* for 10 min at 4°C, unless noted otherwise. Bacterial cryostocks were prepared in LB medium with 20% glycerol and stored at −80°C.

### Isolation of Genomic DNA and Whole Genome Sequencing

*Bartonella henselae* strains grown in BALI medium (isolated from a single colony to avoid a mixture of genomically rearranged subclones) were washed three times with phosphate-buffered saline (PBS; pH 7.2). High molecular weight (HMW) DNA was isolated using the MagAttract HMW DNA kit (Qiagen) and subsequently sheared to ca. 10–12 kb fragments with g-TUBEs (Covaris). The sequencing library was prepared following the Pacific Biosciences (PacBio) protocol for single-molecule real-time (SMRT)bell™ libraries using PacBio^®^ Barcoded Adapters for multiplex SMR^®^ Sequencing. Library samples were size selected using 0.45× AMPure PB beads and sequenced in a single run (i.e., movie and pre-extension time of 20 and 4 h, respectively) on a PacBio Sequel instrument using v3.0 sequencing chemistry, Sequel polymerase v3.0, and an SMRT cell v3 LR tray.

**FIGURE 1:**
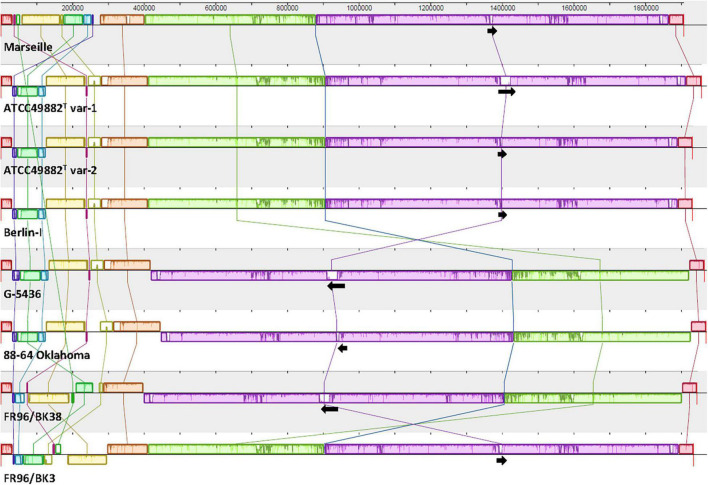
Comparative genome alignment of eight *B. henselae* strains. A genome alignment, visualised via progressiveMAUVE, displays a conserved *B. henselae* genome sequence with few inversions, mostly located in the first ca. 300,000 bp. Genomes are shown as horizontal panels following a black line. Coloured blocks and vertical lines depict localised collinear regions of the genome sequence that align to part of another genome, are homologous, and are internally free from genomic rearrangements. Blocks above or below the horizontal line are in the same or reverse complement orientation compared to the reference genome of *B. henselae* Marseille, respectively. Regions outside blocks lack detectable homology. Inside each block, a similarity profile of the genome sequence is drawn. The height of this profile corresponds to the average level of conservation in that particular region. Complete white areas are not aligned and probably contain sequence elements specific to that strain genome. Black arrows indicate the length, orientation and position of the *badA* island within each genome sequence. The upper scale gives sequence coordinates.

Reads were demultiplexed using Barcoding pipeline on SMRT Link Analysis Services (v5.1.0.26412 and GUI v5.1.0.26411) with a barcode score of ≥26. *De novo* genome assembly was performed using the HGAP 4 pipeline via SMRT Link Analysis Services (v6.0.0.47841, and GUI v6.0.0.47836) with an expected genome size of 2 Mbp, resulting in single contigs (Figure 1). Circular consensus sequences (CCS) reads were computed for the demultiplexed dataset with ≤1 as a number of passes and ≤0.9 as predicted accuracy (Supplementary Table 1).

### Genomic Analysis and Bioinformatic Tools

**FIGURE 2:**
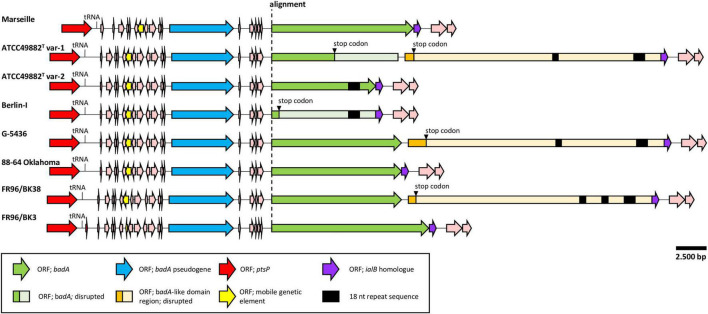
Comparative genetic organisation of the chromosomal *badA* island region. All regions are shown in the same orientation. The conserved upstream ORF (blue) is a *badA* pseudogene containing the typical TAA C-terminal anchor domain. A higher sequence variability is found within the *badA* gene (green), where a premature stop codon is observed in strains ATCC49882*^T^* var-1 and Berlin-I. Strains ATCC49882*^T^* var-1, G-5436, and FR96/BK38 share an exceptionally long ORF (orange), named the *badA*-like domain region that is located downstream of *badA*. These three ORFs also contain the typical TAA C-terminal anchor domain. Additional small ORFs are found between the upstream *badA* pseudogene and *badA* as well as other ORFs flanking the *badA* island (pink). In five displayed *badA* island sequences, two or three unique repeat regions (black rectangle) in either *badA* or in the downstream BadA-like domain region are detected.

Genomes were annotated via both the National Center for Biotechnology Information (NCBI) Prokaryotic Genome Annotation pipeline (Tatusova et al., 2016) and the RASTtk pipeline (Aziz et al., 2008; Brettin et al., 2015). Gene annotation in the *badA* island and flanking up- and downstream regions were manually revised (Figure 2). Correct mapping, assembly, and coverage quality of specific genomic regions were checked *in silico* using CCS reads (≥Q20) uploaded in Minimap2 software (Li, 2018) as a Geneious Prime 2020.0.5 plug-in.

Complete multiple alignment of the *B. henselae* conserved genomic sequences was performed using progressiveMAUVE software (Darling et al., 2004, 2010). ProgressiveMAUVE identifies so-called locally collinear blocks among the various strains that prove to be internally free from genome rearrangements (Figure 1). Genome comparison as per average nucleotide identity (ANI) based on MUMmer (%) was done with the JSpecies Web Server (Kim et al., 2014; Richter et al., 2016; Table 2). Furthermore, potential prophage sequences within the studied *B. henselae* genomes were predicted using Phage Search Tool Enhanced Release (PHASTER) software (Zhou et al., 2011; Arndt et al., 2016). Remote homologues of certain unspecified open reading frames (ORF) were identified in the PDB and Pfam-A databases using the HHpred software (Söding, 2005; Hildebrand et al., 2009; Zimmermann et al., 2018; Steinegger et al., 2019). Further general sequence analyses were consistently performed via SnapGene software (Insightful Science). All mentioned systems and software were run on default parameters.

**TABLE 2:** Genome comparison as per average nucleotide identity (ANI) based on MUMmer (%).

	Marseille	ATCC49882*^T^* var-1	ATCC49882*^T^* var-2	Berlin-I	G-5436	88-64 Oklahoma	FR96/BK38	FR96/BK3
**Marseille**	–	98.83 (97.88)	98.84 (97.61)	98.84 (97.61)	98.84 (97.89)	98.83 (97.89)	98.82 (98.40)	98.91 (99.06)
**ATCC49882^T^ var-1**	98.84 (96.17)	–	99.99 (99.53)	99.98 (99.53)	99.98 (100.00)	99.92 (98.89)	99.37 (98.19)	98.60 (97.36)
**ATCC49882^T^ var-2**	98.85 (96.95)	99.99 (99.99)	–	99.99 (100.00)	99.99 (100.00)	99.92 (99.70)	99.37 (98.17)	98.61 (98.14)
**Berlin-I**	98.85 (96.94)	99.98 (99.99)	99.99 (100.00)	–	99.99 (100.00)	99.92 (99.70)	99.37 (98.17)	98.62 (98.14)
**G-5436**	98.84 (96.17)	99.99 (99.99)	99.99 (99.53)	99.99 (99.53)	–	99.92 (98.90)	99.39 (98.21)	98.61 (97.26)
**88-64 Oklahoma**	98.83 (96.31)	99.92 (99.96)	99.92 (99.72)	99.92 (99.72)	99.92 (99.97)	–	99.39 (97.23)	98.60 (98.38)
**FR96/BK38**	98.82 (96.99)	99.38 (98.45)	99.38 (97.98)	99.38 (97.98)	99.39 (98.45)	99.40 (97.45)	–	98.58 (97.46)
**FR96/BK3**	98.89 (97.75)	98.60 (97.74)	98.61 (97.40)	98.61 (97.40)	98.60 (97.74)	98.60 (97.75)	98.57 (97.58)	–

*The upper value represents the genome comparison as per ANI (%) while the value in parentheses represents the percentage of aligned nucleotides. ANI is visualised with a colour scale gradient ranging from yellow to red (i.e., lower to higher ANI).*

### Generation of a *Bartonella henselae* Marseille *badA* In-Frame Deletion Mutant

A markerless *badA* deletion mutant in *B. henselae* Marseille was generated via a two-step selection process as previously described (Stahl et al., 2015; Weidensdorfer et al., 2016). Primers used in this study are listed in Supplementary Table 2.

Vector pBIISK_*sacB*/*kanR*_UpBadA_DownBadA was constructed by ligating two 1-kb flanking regions from up- and downstream the *badA* gene (Marseille), into the linearised pBIISK_*sacB*/*kanR* plasmid directly downstream of *kanR* via Gibson Assembly^®^ (NEB; Gibson et al., 2009). The first fragment contains a 1-kb upstream non-coding region, as well as 30-bp of 5′-*badA* (amplified using primers FrUp_Fw and FrUp_Rv). The second fragment contains 30-bp of 3′-*badA*, as well as a 1-kb downstream non-coding region (amplified using the primers FrDown_Fw and FrDown_Rv). Plasmid pBIISK_*sacB*/*kanR* was linearised using primers pBIISK_Fw and pBIISK_Rv.

pBIISK_*sacB*/*kanR*_UpBadA_DownBadA was propagated in heat-shock transformed *E. coli* DH5α and subsequently electroporated in *B. henselae* Marseille using a Gene Pulser II electroporator (Bio-Rad) as previously described (Riess et al., 2003). Briefly, ca. 4 × 10^8^ electro-competent *B. henselae* Marseille cells were electroporated with 10 μg of purified plasmid DNA, including 1 μl of TypeOne™ Restriction Inhibitor (Lucigen), and immediately incubated in 1 ml recovery broth for 4 h in a humidified atmosphere at 37°C with 5% CO_2_ while gently shaking (120 RPM).

Transformed bacteria were subsequently incubated for positive selection on KAN-supplemented CBA plates and resulting clones were checked for integration of pBIISK_*sacB*/*kanR*_UpBadA_DownBadA in the genomic DNA by colony PCR using various primer combinations (pBIISK_Fw, pBIISK_Rv, IntegrationA_Fw, IntegrationA_Rv, IntegrationB_Fw, and IntegrationB_Rv). Clones with a correct insertion were transferred onto CBA-plates supplemented with 10% sucrose to facilitate and select for segregation of the integrated suicide vector together with the *badA* gene. Grown colonies were further identified to check for proper vector loss on CBA plates with and without KAN. Finally, correct *badA* deletion was verified by colony PCR, Sanger sequencing (primers in Supplementary Table 2), and Western blotting using rabbit anti-BadA IgG antibodies (see below) using *B. henselae* Marseille as positive control.

### Transmission Electron Microscopy

*Bartonella henselae* strains were grown in BALI medium starting from single colonies and fixed with 4% paraformaldehyde and 2.5% glutaraldehyde (both Electron Microscopy Sciences) in 0.1 M phosphate buffer (pH 7.4) for 90 min at RT. Fixed samples were stored at 4°C until processed either by progressive lowering of temperature (PLT) in dimethylformamide (DMF) and embedding in Lowicryl K4M (protocol adapted from Bayer et al., 1985), or by high-pressure freezing (HPF), freeze substitution (FS) and Epon embedding. Average length of expressed BadA was determined using 24 (for strain FR96/BK38) to 65 (for strain Marseille) bacterial cell images.

For PLT in DMF, fixed bacteria were washed twice in phosphate buffer (1,500 × *g*) and embedded in 12% melted (37°C) gelatine (Merck). Solidified samples were sliced into 1 mm^3^ cubes and fixed in 1% glutaraldehyde for 5 min at 4°C. Samples were dehydrated by gradually increasing the DMF concentration from 30% DMF (in H_2_O) for 30 min at 0°C to 100% DMF for 1 h at −35°C. Lowicryl K4M was infiltrated at −35°C and polymerised by UV. For HPF/FS, fixed bacteria were successively cryofixed in cellulose capillaries in planchettes filled with 1-hexadecene in a high-pressure freezer (Compact 03, Wohlwend), freeze-substituted in 2% osmium tetroxide/0.4% uranyl acetate in acetone, and embedded in Epon. Finally, ultrathin sections were stained with uranyl acetate and lead citrate, and analysed with a Tecnai Spirit electron microscope (Thermo Fisher Scientific) operated at 120 kV.

### Generation of an Anti-BadA Antibody and Western Blotting

Novel rabbit anti-BadA IgG antibodies were developed using isolated BadA proteins derived from the growth culture supernatant of vortexed (2 min) *B. henselae* Marseille. BadA and other large proteins were precipitated by incubating the supernatant in 5% polyethylene glycol 6000 (Carl Roth) o/n at 4°C while slightly shaking, and subsequently collected by centrifugation at 10,000 ×*g* for 1 h at 4°C. The pelleted sample was separated via sodium dodecyl sulphate-polyacrylamide gel electrophoresis (SDS-PAGE) in a single-well 8% gel without prior heat denaturing and was stained o/n with Coomassie Blue R. BadA protein was precisely sliced from the top of the stacking gel and its identity was confirmed via mass spectrometry (see below and Supplementary Figure 1) and used as antigen (ca. 75 μg/injection) for generation of rabbit anti-BadA IgG antibodies (Eurogentec). Rabbit pre-immune serum was used as negative control to verify BadA antibody specificity via Western blotting. Antibodies were further purified to eliminate unspecific antibody background reaction by pre-adsorption with *B. henselae* Marseille ΔBadA-T (5 × 10^9^ cells/ml) for 2 h at RT, while shaking (900 RPM).

BadA protein expression was analysed via Western blotting using the rabbit anti-BadA IgG antibodies (this study). Whole cell *B. henselae* strains were grown in BALI medium for three days, collected by centrifugation, and subsequently prepared by incubation in Laemmli sample buffer (Sigma-Aldrich) for 10 min at 95°C. Denatured proteins were separated via SDS-PAGE on a 4–15% gradient gel (Bio-Rad) and transferred to nitrocellulose membranes for 1 h at 300 mA in Towbin transfer buffer (10% glycerol). Blotted membranes were incubated o/n at 4°C with rabbit anti-BadA IgG antibodies (1:4,000) followed by a second incubation for 90 min at RT with HRP-conjugated swine anti-rabbit IgG antibodies (1:2,000; Agilent-Dako). Detected proteins were developed using SuperSignal West Pico PLUS Chemiluminescent Substrate (Thermo Scientific) and analysed on a ChemiDOC XRS + system (Bio-Rad) with ImageLab V6.0.1. software (Bio-Rad).

### Mass Spectrometry

The Coomassie Blue R-stained SDS-gel fragment was cut into small pieces and subsequently prepared for mass spectrometry (MS). Briefly, proteins were denatured (8 M urea and 100 mM ammonium bicarbonate) and 5 mM tris(2-carboxyethyl)phosphine hydrochloride was added to reduce the amount of disulphide bonds. 10 mM iodoacetamide was added for alkylation in a dark room. Finally, the samples were diluted in 100 mM ammonium bicarbonate and 0.5 mg/ml sequencing-grade trypsin (Promega) was added to digest the proteins into peptides. Peptides were analysed on a Q Exactive HFX connected to an Easy-nLC 1200 instrument (Thermo Scientific).

### Immunofluorescence Microscopy

*Bartonella henselae* BadA surface expression was assessed via immunofluorescence microscopy testing using rabbit anti-BadA IgG antibodies (this study). *B. henselae* strains grown in BALI medium were resuspended in PBS, airdried on glass microscopy slides (KNITTEL StarFrost®), and fixed with 3.75% paraformaldehyde for 10 min at 4°C. Fixed bacteria were stained with rabbit anti-BadA IgG antibodies (1:400) and subsequently incubated with goat IgG anti-rabbit IgG conjugated to Alexa 488 (1:200; Dianova), both for 1 h at RT. Bacterial DNA was stained with 4′,6-diamidino-2-phenylindole (1 μg/ml; DAPI; Merck) for 10 min at 4°C. All incubation steps were performed in a humid chamber and followed by three washes with PBS. Slides were mounted with fluorescence medium (Dako), air-dried, and analysed with a Zeiss Axio Imager 2 microscope equipped with a Spot RT3 microscope camera (Diagnostic Instruments Inc.) operated by VisiView V.2.0.5 (Visitron Systems).

### Binding of *Bartonella henselae* to Extracellular Matrix Proteins

The binding of *B. henselae* to ECM proteins was tested *in vitro* using an enzyme-linked immunosorbent assays (ELISA). Briefly, 1 μg of human plasma fibronectin (Fn, Sigma-Aldrich) or human collagen-I (Col-I, Merck) was coated o/n at 4°C onto Nunc Maxisorp flat-bottom 96-wells (Thermo Scientific). Wells were blocked with 2% w/v bovine serum albumin (BSA; Sigma-Aldrich) in washing buffer (0.05% v/v Tween 20 in PBS) and incubated for 90 min at 37°C. *B. henselae* strains grown in BALI medium were resuspended in PBS, added to the wells (ca. 2 × 10^8^ cells), and incubated for 2 h at 37°C. Bound bacteria were detected using anti-*B. henselae* IgG antibodies (1:1,000 in blocking buffer; Kempf et al., 2000) and swine anti-rabbit HRP (1:2,000 in blocking buffer). Samples were developed using 3,3′,5,5′-tetramethylbenzidine liquid substrate (TMB; Sigma-Aldrich) for ca. 1 min and the reaction was stopped with 1M HCl. The resulting absorbance was measured at 450 nm using a microplate Sunrise-Basic™ reader (TECAN). All 96-well plates were sealed to prevent evaporation during incubation steps, and were each time immediately followed by three washes in wash buffer. Assays were done in triplicate.

### Statistics

Statistical analyses were performed using one-way ANOVA testing on Prism V7.04 (GraphPad Software) assuming parametric data distribution. A value of *p* < 0.01 was considered statistically significant.

## Results

### Long-Read Sequencing Demonstrates a Conserved *Bartonella henselae* Genome With Only Few Divergences in Genomic Organisation

*Bartonella henselae* strains and substrains from our strain collection were included in this study for which the origin was traceable. Eight complete and single contig genomes of different *B. henselae* (sub)strains (Table 1) were generated using next-generation long-read Pacific Biosciences SMRT sequencing. The Phred quality (Q) score of the CCS reads ranges from Q32 (99.94% accuracy) to Q34 (99.96% accuracy) for reads filtered to have a score above Q20. In case of the strains Marseille and ATCC49882*^T^* var-1, an overall Q-score with an accuracy of 98.7% and 98.9% was observed, respectively. The genome of *B. henselae* consists of a single circular chromosome with a size that ranges from ca. 1.91 Mbp (Marseille) to 1.97 Mbp (88-64 Oklahoma) and a low GC-content of 38%. All analysed genomes start with the housekeeping gene *gltA* (sense) and genes encoding for *Bartonella* adhesin A (BadA), the Trw locus, the VirB/D4 locus, and Pap31 (Lu et al., 2013) were identified in all strains. An overview of general sequencing parameters and genome features is given in Supplementary Table 1.

Whole-genome comparison as per ANI results in a score ranging from 98.57 to 99.99% indicating a low intra-species genome diversity (Table 2). A multiple genome alignment using progressiveMAUVE shows a conserved core genome with only few divergences in genomic organisation (Figure 1). Most genomic rearrangements are located in the first ca. 300,000 bp where a dispersed array of collinear and inversed regions is observed. This region corresponds to a previously described type II secretion system island including phage genes potentially linked to genomic variability (Alsmark et al., 2004; Engel and Dehio, 2009). In that same variable region, one or two (depending on the strain) incomplete but potential prophage sequences are predicted using PHASTER software (data not shown).

Further downstream from ca. nucleotide (nt) 400,000 to 1,850,000, a major genomic inversion around two adjacent collinear regions is identified in strains G-5436, 88-64 Oklahoma, and FR96/BK38 (Figure 1). Both sides of the inversion are characterised by a copy of the highly conserved *tuf* gene (elongation factor Tu; EF-Tu), that is flanked by either *fusA* (elongation factor G; EF-G), or genes encoding for ribosomal and transcription-related proteins possibly forming a transcriptional unit.

Based on the ANI results (Table 2) and the multiple genome alignment (Figure 1), strains ATCC49882*^T^* var-1, ATCC49882*^T^* var-2, Berlin-I, G-5436, 88-64 Oklahoma, and FR96/BK38 are classified as a separate *B. henselae* subgroup. Both strains Marseille (genotype II) and FR96/BK3 appear to be genetically distinct.

### The *badA* Genomic Island Consists of a Variable *badA* Gene Flanked by *badA* Pseudogenes and Conserved Up- and Downstream Regions

A noteworthy low similarity profile is observed around the location of the *badA* gene indicating a high(er) sequence variability in the otherwise rather conserved purple region (Figure 1). The chromosomal region containing *badA* (Figure 2 and Supplementary Table 3) demonstrates a variable sequence length of *badA* (8.7 to 13.2 kb) among the various strains. In strains ATCC49882*^T^* var-1 and Berlin-I a frameshift mutation causes a premature stop codon. The *badA* gene is preceded by a conserved *badA* pseudogene and, in case of strains ATCC49882*^T^* var-1, G-5436, and FR96/BK38 followed by a large (ca. 21 kb) ORF disrupted by a premature stop codon with a highly similar domain organisation to *badA*. Depending on the *B. henselae* strain, four or five ORFs are identified in between the *badA* pseudogene and the *badA* gene. The ORF directly upstream of *badA* annotates as a surface protein and shares homology with TAAs, for strains Marseille and FR96/BK3, this applies to the last two ORFs.

In strains ATCC49882*^T^* var-1, ATCC49882*^T^* var-2, Berlin-I, G-5436, and FR96/BK38, a unique repeat sequence is observed in the *badA* island with a repeat unit length of 18 nt (5′-ART GGC GGA AGC AAY GGY-3′), and a number of repeat units of either 26 or 51. The authenticity of this sequence motif was verified by PCR (primers Repeat_Fw and Repeat_Rv) and Sanger sequencing (data not shown). Interestingly, only in strain ATCC49882*^T^* var-2 this repeat sequence is located in the expressed BadA protein.

The *badA* island is flanked up- and downstream by a conserved gene array in all eight *B. henselae* strains. Genome annotation of this genomic island and flanking regions by both RASTtk and NCBI pipelines were individually checked *in silico* to ensure accurate and complete gene annotations.

The following genes which are located in the region ca. 25 kb upstream of the *badA* pseudogene potentially influence BadA expression: (i) an iron response regulator (*irr*; 504 bp) showing high sequence similarity to a ferric uptake repressor-like protein (Fur; for example with *irr* of *B. bacilliformis* ATCC 35685) located ca 23.5 kb upstream of the *badA* island, (ii) a resistance-nodulation-cell division (RND) efflux inner membrane transporter subunit (3,135 bp), and (iii) several ribosomal subunit proteins. In addition, ca. 10 kb upstream of the *badA* pseudogene, a conserved phosphoenolpyruvate-protein phosphotransferase gene (*ptsP*; 2,505 bp) is present. Further, a mobile genetic element (480 bp) is located ca. 3.4 kb upstream of the *badA* pseudogene and shows high sequence similarity among all strains (>99%), except for strain FR96/BK3 that displays a shorter ORF (201 bp) due to a premature stop codon. The deduced protein shows a high degree of sequence similarity with three proteins from the AAA + ATPase (ATPase associated with diverse cellular activities) superfamily that are all, among other functions, involved in DNA transposition, recombination-dependent replication, and damage repair (i.e., the MuB transposition protein, the RuvB-like protein 2, and the replication-associated recombination protein A). Following the mobile genetic element, a peculiar drop in sequence similarity is observed for strain Marseille, caused by a ca. 1.2 kb deletion. Overall, while strains ATCC49882*^T^* var-1, ATCC49882*^T^* var-2, Berlin-I, G-5436, and 88-64 Oklahoma are fully identical in the region (ca. 25 kb) upstream of the *badA* island, several small genomic variations (e.g., point mutations and short deletions or insertions) are observed in the strains Marseille and FR96/BK3, indicating more frequent recombination events.

Similar to the upstream region, strains ATCC49882*^T^* var-1, ATCC49882*^T^* var-2, Berlin-I, G-5436, and 88-64 Oklahoma show an identical downstream region (ca. 20 kb). Small genomic variations (e.g., point mutations and short deletions or insertions) appear only in strains Marseille, FR96/BK3 and occasionally in FR96/BK38 (not depicted). The immediate downstream ORF (573 bp) of *badA* (or the downstream *badA*-like domain region in case of strains ATCC49882*^T^* var-1, G-5436, and FR96/BK38) shows a high sequence similarity with the invasion associated locus B (*ialB*) gene of *Bartonella* spp. A frameshift mutation in strains Marseille and FR96/BK3 shifts the transcription start site of this *ialB* homologue downstream with 16 bp. The proximity of the *ialB* homologue to *badA* suggests a single operon and subsequent protein association. In addition, the *B. henselae ialB* gene (Deng et al., 2016) is found ca. 13.5 kb downstream of the *badA* island.

### The *badA* Pseudogene Shows Low Intraspecies Variations and Has a Similar Architecture to *badA*

A *badA* pseudogene is found upstream of *badA* in all eight *B. henselae* strains (Figure 2 and Supplementary Table 3) showing a similar length (5.2 kb to 5.4 kb) and a high sequence similarity (80–100%). Strains ATCC49882*^T^* var-1, ATCC49882*^T^* var-2, Berlin-I, G-5436, and 88-64 Oklahoma show an identical *badA* pseudogene sequence, confirming their close relation. The putative C-terminal region of the corresponding protein (ca. 550 aa) including the putative anchor domain is identical among all eight strains analysed in this study.

The *badA* pseudogene sequence is considerably shorter than the *badA* gene but translates to a similar TAA architecture, including a head domain, a neck/stalk region, and an anchor domain (not shown). Certain regions in the *badA* pseudogenes align in sequential but interspersed order to regions in the *badA* genes with similarities ranging from 50 to 83%. The highest sequence similarities are found in the sequence for the anchor domain. Thus far, expression of the *badA* pseudogene is not analysed yet. The *badA* promoter is predicted to be located in a region ca. 350 bp upstream of the transcription start site (Okaro et al., 2020), and thus downstream of the *badA* pseudogene.

### Three *Bartonella henselae* Strains Contain a Large Downstream *badA*-Like Domain Region

Three *B. henselae* strains (ATCC49882*^T^* var-1, G-5436, and FR96/BK38) share an enormous *badA*-like domain region directly downstream of *badA* (Figure 2 and Supplementary Table 3). This region encodes the typical C-terminal anchor domain sequence, as well as numerous repeat domains forming tandem arrays of repeat units, as similarly observed in *badA*. However, no distinguishable head domain is observed. A single bp mutation causes a premature stop in the otherwise perfect ORF coding for a *badA*-like domain region of either 7,128 aa (for strains ATCC49882*^T^* var-1 and G-5436) or 6,834 aa (for strain FR96/BK38). ATCC49882*^T^* var-1 and G-5436 share an identical *badA*-like domain region (with the exception of 2 bp), while a double deletion of ca. 600 bp in strain FR96/BK38 results in a shorter ORF and three instead of two unique 18 nt repeat sequence motifs (see above).

### *Bartonella* Adhesin A Shows a High Intraspecies Variability Linked to Recombination Events

All eight studied *B. henselae* genomes include a *badA* gene (of which two are disrupted by an early stop codon) within their respective *badA* genomic island (Figure 2) and each of the deduced proteins show the characteristic TAA architecture consisting of an N-terminal head domain, a long and repetitive neck/stalk region, and a conserved C-terminal anchor domain (Szczesny and Lupas, 2008). Despite their overall similarities, a great variety in length, expression, and neck/stalk domain order is observed.

**FIGURE 3:**
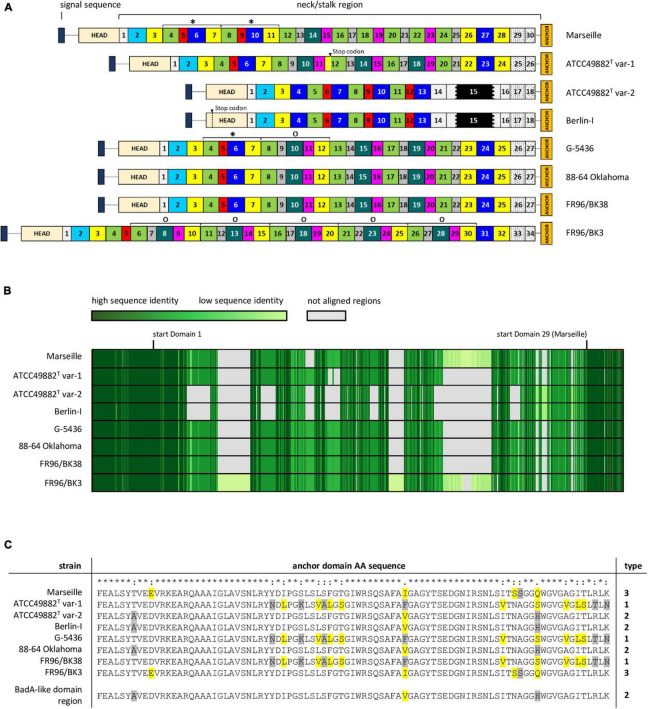
Schematic comparison and aa-alignment of deduced BadA protein sequences among eight *B. henselae* strains. **(A)** BadA subdivided in numerous predicted domains based on the typical neck sequences demonstrates the high number of repeated motifs within a single protein, as well as among the various tested strains (* and ^O^). Each domain colour represents a domain category that show mutual protein sequence similarity. The neck/stalk region, where domains are presumably multiplied, deleted, or recombined via recombination events, is diverse among the strains. Due to a frameshift mutation, a stop codon is observed in BadA domain 12 of strain ATCC49882*^T^* var-1, and in the head domain of strain Berlin-I. The black region within domain 15 in strains ATCC49882*^T^* var-1 and Berlin-I represents the unique 18 nt-long repeat sequence motif. **(B)** BadA protein sequence alignment (MUSCLE) shows a conserved N-terminal (ca. first 1,000 aa) and C-terminal region (ca. last 330 aa) with a higher diversity observed in the BadA neck/stalk region. **(C)** Protein sequence alignment (MUSCLE) of the anchor domain reveals the presence of three different types (1, 2, and 3). Highlighted aa show differences among aligned sequences that share similar biochemical properties (yellow), or that do not (grey).

The number of distinguishable domains in the neck/stalk region ranges from 18 in strains ATCC49882*^T^* var-2 and Berlin-I, to 34 in strain FR96/BK3, and domain sizes vary between 68 aa and 147 aa (Figure 3A). Among the studied strains, all BadA domains in the neck/stalk region fall into distinct categories based on pairwise similarities in protein sequence (visualised by similar colours), except for domain 14, 15, and 16 in strains ATCC49882*^T^* var-2 and Berlin-I. This domain composition illustrates the repetitive nature of the BadA protein and the variability among the different *B. henselae* strains.

The repetitive *badA* structure complicates gene assembly based on short-read sequencing data. Using long-read sequencing, the full *badA* gene of the well-studied strain Marseille was covered in single reads, resulting in a 11,922 bp gene (Supplementary Table 3). The expressed protein consists of 3,973 aa (including the signal sequence of 47 aa) containing 30 neck/stalk domains (GenBank: MK993576.1). As such, two repeated motifs, each consisting of four domains, were previously overlooked (GenBank: DQ665674.1) by Sanger sequencing (Riess et al., 2004).

The neck/stalk region of *badA* among the *B. henselae* strains is highly variable compared to the more conserved head and membrane anchor sequences (Figure 3B). Furthermore, three types of *badA* anchor domain sequences (89 aa) were detected (Figure 3C). Type 1 is observed in strains ATCC49882*^T^* var-1, G-5436, and FR96/BK38 and shows high sequence similarity with the other two anchor domain types (37 and 44 bp differences, respectively). Type 2 is present in the strains Berlin-I, ATCC49882*^T^* var-2, and 88-64 Oklahoma and is identical to the anchor domain of the downstream *badA*-like domain region. Type 3 is found in strains Marseille and FR96/BK3 and shows 16 bp mutations (resulting in 6 aa differences) compared to type 2.

These observations suggest the occurrence of recombination events in *badA* and the *badA* island in general. For instance, strains ATCC49882*^T^* var-1, G-5436, 88-64 Oklahoma, and FR96/BK38 show similar *badA* sequence arrangements, while strains Berlin-I and ATCC49882*^T^* var-2 display a considerably shorter and divergent *badA* sequence, including the unique 18 nt-long repeat sequence motif within domain 15 (Figure 3A). The *badA* gene of the latter two strains seems the result of a fusion between the highly conserved N-terminal *badA* (up to 69 bp before the start of domain 4) and a formerly present C-terminal *badA*-like domain region (last 6,360 bp), as seen in strains G-5436 and ATCC49882*^T^* var-1, showing an overlap of 39 bp. A similar recombination in the *badA* island in strain 88-64 Oklahoma connecting the end of *badA* domain 26 with the last 697 bp of the *badA*-like domain region may have resulted in this current *badA* gene. Moreover, it is likely that a recombination event including a former *badA*-like domain region anchor sequence and part of the N-terminal *badA* sequence previously resulted in a common *badA* gene for strains Marseille and FR96/BK3. Both strains probably underwent further gene rearrangements in their respective *badA* genes. Indications thereof can be found in multiplied neck/stalk domain segments seen in BadA of strain G-5436 which are found to be repeated two and five times in BadA of strains Marseille and FR96/BK3, respectively (Figure 3A).

### Surface Expression and Length of BadA Variants Correlate With Their Respective Genomic Sequence

Exact strain origin, passage number, BadA expression status, and BadA length are important characteristics often unknown or omitted in studies involving *B. henselae* (Table 1). Therefore, we performed a detailed analysis of the BadA surface expression status of eight *B. henselae* strains. Based on the long-read sequencing data, frameshift mutations in strain ATCC49882*^T^* var-1 (caused by the deletion of a 262-bp region, at position 5,266 bp from the start codon of *badA*) and in strain Berlin-I (caused by a single bp deletion; at position 584 bp from the start codon of *badA*) both result in a premature stop codon (Figure 2). The *badA* genes of the other strains appeared intact.

**FIGURE 4:**
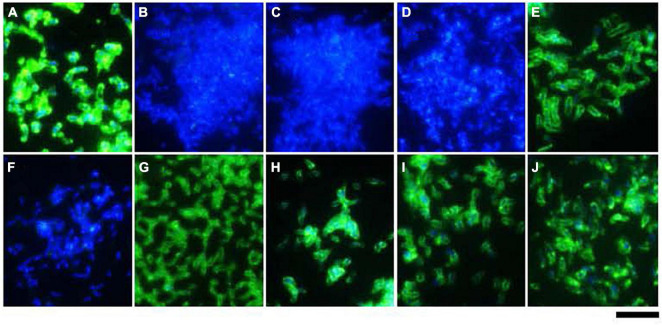
Surface expression of BadA in various *B. henselae* strains (immunofluorescence microscopy). Surface expression of BadA was analysed via immunofluorescence microscopy using specific anti-BadA IgG antibodies (green). Bacterial DNA was counterstained using DAPI (blue). The illustrated *B. henselae* strains are **(A)** Marseille, **(B)** Marseille ΔBadA-T, **(C)** Marseille ΔBadA-D, **(D)** ATCC49882*^T^* var-1, **(E)** ATCC49882*^T^* var-2, **(F)** Berlin-I, **(G)** G-5436, **(H)** 88-64 Oklahoma, **(I)** FR96/BK38, and **(J)** FR96/BK3. Expression is observed for strains Marseille, ATCC49882*^T^* var-2, G-5436, 88-64 Oklahoma, FR96/BK38, and FR96/BK3, detected by the characteristic green halo. Strains ATCC49882*^T^* var-1 and Berlin-I do not express BadA, nor do the negative control strains Marseille ΔBadA-T and Marseille ΔBadA-D. Scale bar: 5 μm.

**FIGURE 5:**
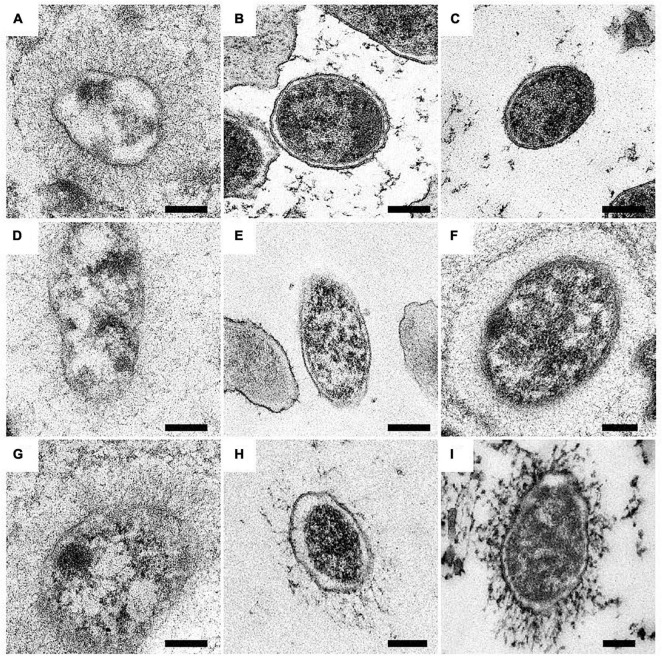
Surface expression of BadA in various *B. henselae* strains (transmission electron microscopy). Representative images of *B. henselae* **(A)** Marseille, **(B)** Marseille ΔBadA-T, **(C)** ATCC49882*^T^* var-1, **(D)** ATCC49882*^T^* var-2, **(E)** Berlin-I, **(F)** G-5436, **(G)** 88-64 Oklahoma, **(H)** FR96/BK38, and **(I)** FR96/BK3 are visualised with TEM. Around 30-50 TEM images of bacterial cells per strain were analysed for determination of the phenotype. Expression of BadA is observed for strains Marseille, ATCC49882*^T^* var-2, G-5436, 88-64 Oklahoma, FR96/BK38, and FR96/BK3 but not for strains ATCC49882*^T^* var-1 and Berlin-I, nor for the negative control strain Marseille ΔBadA-T. For technical reasons, samples were prepared by both PLT in DMF and K4M embedding (e.g., Marseille, ATCC49882*^T^* var-2, G-5436, and 88-64 Oklahoma), and HPF/FS and Epon embedding (e.g., Marseille ΔBadA-T, ATCC49882*^T^* var-1, Berlin-I, FR96/BK38, and FR96/BK3). Scale bar: 200 nm.

Expression of BadA was assessed using immunofluorescence (Figure 4) and transmission electron microscopy (TEM; Figure 5). Transposon mutants Marseille ΔBadA-T and Marseille ΔBadA-D were used as negative controls. Surface-expressed BadA was clearly shown for strains Marseille, ATCC49882*^T^* var-2, G-5436, 88-64 Oklahoma, FR96/BK38, and FR96/BK3. Strains ATCC49882*^T^* var-1 and Berlin-I do not express BadA, similar to both negative control strains. These data were corroborated by TEM where two different preparation methods (PLT in DMF and HPF/FS) were used to achieve optimal visualisation of BadA.

**FIGURE 6:**
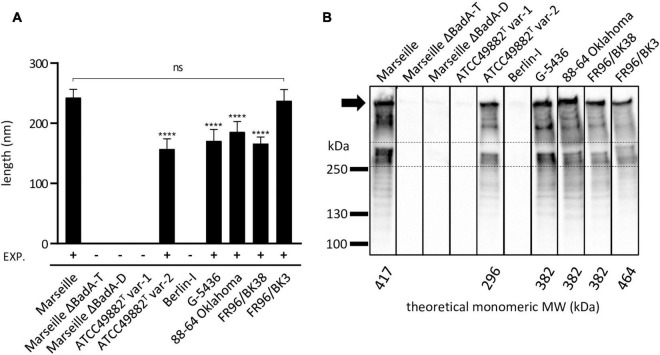
Length analysis of surface-expressed BadA and their corresponding molecular weight of *B. henselae* strains. **(A)** The average length of expressed BadA was measured using 30-50 TEM images of bacterial cells per strain. The individual average BadA fibre lengths of strains ATCC49882*^T^* var-2 (157 nm), G-5436 (171 nm), 88-66 Oklahoma (186 nm) and FR96/BK38 (166 nm) are significantly shorter (****p < 0.0001) compared to those of strains Marseille (243 nm) and FR96/BK3 (238 nm). The length of the last two strains do not differ significantly (ns). Strains ATCC49882*^T^* var-1, Berlin-I, Marseille ΔBadA-T and Marseille ΔBadA-D do not express BadA. EXP.: expression of BadA. **(B)** BadA expression of the various *B. henselae* strains is visualised by Western blotting. The band between the dashed lines is considered monomeric BadA protein. Marseille and FR96/BK3 display a higher MW band around the predicted monomeric BadA protein. The numerous lower MW-bands are considered degradation products of the high MW BadA protein. The uppermost band is presumably trimeric BadA protein stuck in the pocket of the gel (black arrow). Bands in between the trimeric and monomeric protein are also considered degradation products of trimeric BadA. All strains were analysed on a single nitrocellulose blot, the order of columns has been rearranged *in silico*.

The average length of BadA of the particular *B. henselae* strains was analysed from transmission electron microscopy (TEM) images (Figure 6A). The lengths of the BadA fibres are in accordance with their identified *badA* gene lengths. Strains Marseille (BadA; 3,973 aa) and FR96/BK3 (BadA; 4,407 aa) express the longest fibres measuring 243 and 238 nm on average, respectively, while strain ATCC49882*^T^* var-2 (BadA; 2,920 aa) presents the shortest fibres with an average length of 155 nm. Strains G-5436 (BadA; 3,641 aa), 88-64 Oklahoma (BadA; 3,643 aa), and FR96/BK38 (BadA; 3,641 aa) display comparable fibres with average lengths of 171, 186, and 166 nm, respectively.

Finally, the molecular weights (MW) and expression of the various BadA proteins were visualised via Western blotting using rabbit anti-BadA IgG antibodies (Figure 6B). Blotting results are in line with immunofluorescence and electron microscopy results, as well as with MW predictions of the monomeric BadA proteins (ranging from 296 to 464 kDa). Although the resolution of SDS-PAGE does not allow for a precise MW quantification of the trimeric BadA protein, strains Marseille and FR96/BK3 show a slightly larger monomeric BadA protein (417 and 464 kDa, respectively) compared to the other strains. The uppermost detected protein is presumed to be trimeric BadA that is unable to travel down in the gel because of its enormous size, heat stability, and incomplete denaturation (Grosskinsky et al., 2007).

### The Surface Expression of BadA Determines Extracellular Matrix Binding Independent of Strain Specific Domain Architecture

**FIGURE 7:**
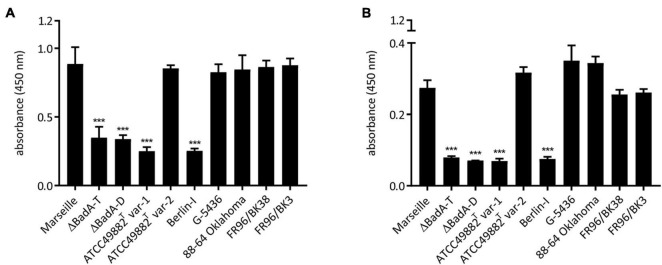
Adhesion of *B. henselae* strains to extracellular matrix proteins. Binding of the various *B. henselae* strains to **(A)** human plasma Fn and **(B)** human Col-I. Wells were coated with Fn and Col-I and adherent bacteria were quantified via whole cell ELISA (see section “Materials and Methods”). BadA-expressing strains show a significantly higher binding to human plasma Fn and human Col-I compared to strains lacking BadA expression. Statistical significance was determined using one-way ANOVA testing (***p < 0.001).

The biological function of the varying BadA proteins in binding of *B. henselae* to various matrix proteins was assessed. For this purpose, bacterial adhesion to human plasma Fn and human Col-I was quantified via a whole-cell ELISA using anti-*B. henselae* IgG antibodies. Surface-expressed BadA proteins clearly mediate bacterial binding to human plasma Fn and human Col-I (Figure 7). A significantly lower adhesion was observed for the BadA-deficient strains ATCC49882*^T^* var-1, Berlin-I, and both negative control strains Marseille ΔBadA-T and Marseille ΔBadA-D. In contrast, ECM adhesion was detected for all strains that express BadA naturally, independent of their individual BadA domain composition, repeats, or various BadA arrangements.

## Discussion

Adhesion to cells is the first and most decisive step in the course of any infection. TAAs represent a major class of pathogenicity factors in Gram-negative bacteria and are defined by their homologous C-terminal membrane anchor (Linke et al., 2006). The best examined TAA is the *Yersinia* adhesin A (YadA) of *Yersinia enterocolitica*, and despite its relatively short gene sequence (1.3 to 1.5 kb) and small fibre length (ca. 23 nm), it is seen as the prototypical TAA (El Tahir and Skurnik, 2001). Examples of other well-studied TAAs are the *Acinetobacter* trimeric autotransporter (Ata) of *A. baumannii* (Weidensdorfer et al., 2019) and the *Neisseria* adhesin A (NadA) of *N. meningitidis* (Comanducci et al., 2002). TAAs share a modular and repetitive domain composition (head, neck, stalk, and membrane anchor domains) (Szczesny and Lupas, 2008) and the underlying repeats on the DNA level suggest easy gene rearrangements via recombination, presumably driven by genetic adaptation to alternating host environments (Lindroos et al., 2006). TAAs of the genus *Bartonella* stand out because of their variability in length, high conservation within the genus, and the presence of multiple gene variants within one genome. The extensive number (30) of repetitive neck/stalk domains in BadA of *B. henselae* Marseille makes this region an excellent “toolbox” for TAA evolution (Figure 3A).

The presence of numerous prophages and genomic islands including both short tandem repeats and longer repeats (such as the *badA* island of *B. henselae*) often result in sequencing and assembly errors. Long-read WGS techniques overcome these issues (Koren et al., 2013; Tørresen et al., 2019). Accordingly, we demonstrate that the length of *badA* from strain Marseille is 11,922 bp (Supplementary Table 3), instead of the earlier determined 9,249 bp (Riess et al., 2004). In addition, long-read sequencing of the *badA* gene from seven other *B. henselae* strains confirmed the previously described varying length of the neck/stalk region among the various *B. henselae* strains (Figure 3B; Riess et al., 2007).

All sequenced *B. henselae* strains show a conserved genome with a high intra-species genome sequence similarity. Divergences are mostly observed in a 300 kb region that includes a defined type II secretion system island and contains various phage genes (Engel and Dehio, 2009). Within this region which contains highly dynamic stretches, genomic inversions were identified (e.g., around the duplicated *tuf* gene and multiple copies of rRNA operons). This prophage region presumably drives diversification and dispersion of specific host-adaptability genes throughout the *B. henselae* population (Anderson et al., 1994; Lindroos et al., 2006; Harvey et al., 2019). Such diverse recombination events make correct species typing or phylogenetic analysis challenging.

Two *B. henselae* genotypes have been described so far. Genotype I is believed to be more associated with human infections, while genotype II appears to outcompete genotype I during bloodstream infections in cats (Huwyler et al., 2017). Strains ATCC49882*^T^* var-1, ATCC49882*^T^* var-2, Berlin-I, G-5436, and 88-64 Oklahoma are all human isolates with high pairwise genome sequence identity of >99.9% (Table 2). In addition, three of these strains are known variants of the type-strain ATCC49882*^T^* Houston-I (Table 1). Based on our data, we suggest to classify these five strains as genotype I. Strain Marseille shows a comparatively lower genome sequence identity (ca. 98.8%) to these five strains (Table 2) and demonstrates that genotype II strains are not exclusively isolated from cats. Compared to the genotype I group, both *B. henselae* cat isolates in this study (strains FR96/BK38 and FR96/BK3) do not show a high pairwise genome sequence identity (98.6%). We propose that the classification of *B. henselae* strains into particular genotypes cannot be solely attributed to the source of isolation. This is in accordance with findings that new genetic variants of *B. henselae* frequently emerge *in vivo* (Iredell et al., 2003; Berghoff et al., 2007).

Based on the various hypothesised rearrangements within the defined *badA* island sequence among the different *B. henselae* strains, including the downstream *badA*-like domain region and the small variations in the BadA anchor domain aa-sequence, we postulate that a series of recombination events has led to the formation of at least three phylogenetically distinct groups of *B. henselae* (Figure 3C). We propose strain G-5436 to be the evolutionary ancestor of the strains in this study because of its intact *badA* sequence and its present (and longer) downstream *badA*-like domain region (compared to strain FR96/BK38). Future classification and bacterial genotyping of *B. henselae* strains should be supported by either long-read WGS results or a detailed analysis of the *badA* island.

As known so far, *badA* gene expression seems to be regulated by (i) the general stress response system (Tu et al., 2016), (ii) the *Bartonella* regulatory transcript in combination with the transcriptional regulator protein (Tu et al., 2017), and (iii) the BatR/S two-component system (Quebatte et al., 2010). These systems are influenced by the extracellular environment, correlating with frequently alternating host conditions (e.g., temperature and pH). The close vicinity of a predicted ferric uptake repressor-like protein (Fur) to *badA* suggests a repressed *badA* expression of *B. henselae* while residing in the iron-saturated flea gut environment. In mammal hosts where free heme as an iron source is rare, upregulation of *badA* might facilitate initial adhesion and subsequent infection (Battisti et al., 2007). An upstream gene (*ptsP*) that is part of a phosphotransferase (PT) system, controls among others the carbohydrate flow in bacteria and is involved in virulence gene expression related to nutrient availability (Bier et al., 2020). An *ialB* homologue of *B. henselae* is located immediately downstream of the *badA* genomic island. It has been described that IalB plays a role in erythrocyte invasion of *Bartonella* species (Coleman and Minnick, 2001; Deng et al., 2016). However, and in contrast to *badA*, *ialB* expression is upregulated by a low pH or decreasing temperatures (Coleman and Minnick, 2003; Okaro et al., 2017). A similar operon configuration involving a TAA gene associated with *ialB* is observed for the *Brucella abortus* trimeric autotransporter adhesin (BatA; Rahbar et al., 2020). The functional relationship of *badA* with Fur, the PT system, and the IalB homologue remains to be explored.

Microscopy and protein expression analyses validate the long-read sequencing results and confirm that all strains express a surface-expressed BadA, except for strains ATCC49882*^T^* var-1 and Berlin-I due to individual frameshift mutations (Figures 4–6). The length of surface-expressed BadA was determined from TEM-images and correlates with the *badA* gene length of the respective *B. henselae* strain. The slight variation between the measured fibre lengths of surface-expressed BadA from *B. henselae* strains G-5436, 88-64 Oklahoma, and FR96/BK38, despite having similar *badA* gene lengths, might derive from structural changes due to the TEM-processing, such as partial protein unfolding (Figure 6 and Supplementary Table 3).

BadA expression was previously shown to be crucial for endothelial cell infection and induction of a proangiogenic response (Riess et al., 2004) while negatively affecting VirB/D4-dependent host cell invasion via “invasome” formation (Lu et al., 2013). Binding of *B. henselae* to ECM proteins human plasma Fn and Col-I also depends on the expression of BadA rather than the observed differences in protein length (Figure 7). The binding of BadA to Fn has been previously attributed to the passenger domain, while the head domain itself was described to be sufficient for collagen binding. In addition, it was demonstrated that a critical length of surface-expressed BadA (40 nm) was necessary to detect binding of *B. henselae* to Fn (Kaiser et al., 2008, 2012). Plasma Fn which is present in blood and other fluids, is a major component of the fibrin clot in early wound healing responses and an important glycoprotein in the ECM. Col-I is the most abundant collagen in the human body, especially in the dermis, and represents a major binding partner for TAAs (Łyskowski et al., 2011; Vaca et al., 2019). In case of a cat scratch or bite in the human dermis, both ECM proteins might thus be an ideal BadA binding partner in the course of infection of *B. henselae*.

The frequent transitions of *B. henselae* from the cat flea’s gut to cats and accidental human hosts might require efficient and quick adaptation strategies. In contrast to other variable regions, the *badA* island has previously not been predicted nor described as a prophage or defined genomic island (Engel and Dehio, 2009). The observed heterogeneity in the *badA* island between different *B. henselae* strains might be the result of evolutionary selection for various beneficial traits such as host-specific colonisation and immune escape. We postulate a shuffling mechanism comparable to phase variation that capitalises on the extensive number of repeats in the *badA* island making this region an excellent “toolbox” for TAA evolution. Combinatorial reshuffling of similar TAA repeats as a mechanism to quickly adapt these immunodominant adhesins has been proposed previously (Szczesny et al., 2008). The highly repetitive *badA* neck/stalk region might facilitate site-specific recombination or slipped-strand mispairing within the *badA* island that reshuffles *badA*, the neighbouring *badA* pseudogenes, and possibly involves the peculiar 18 nt-long repeat sequence motif. The protein structure of this translated 18 nt-long repeat sequence might mimic collagen-like triple helixes which would fit the general TAA structure. Similar phase variation mechanisms were demonstrated earlier for *N. meningitidis* (Bentley et al., 2007). A potential candidate that mediates these recombination events is the identified mobile genetic element protein upstream of the *badA* island. This ORF shows homology with three different AAA + ATPases (i.e., MuB, RuvB-like protein 2, and RarA) that are all involved in DNA recombination. The presence of *B. henselae* isolates lacking BadA expression due to a frameshift mutation (e.g., strain ATCC49882*^T^* var-2 and Berlin-I) or the loss of BadA expression following multiple passages outside a natural host, caused by the deletion of an 8.5 kb genomic fragment (Kempf et al., 2001; Riess et al., 2004), strengthens our hypothesis of existing phase-on and phase-off phenotypes (Anderson and Neuman, 1997; Kyme et al., 2003; Lu et al., 2013). Because of the unknown passage number of the respective bacterial strains (before arriving to our laboratory) we cannot fully rule out that such recombination events might have occurred while cultivating the bacteria under laboratory conditions. However, regaining the ability to express BadA was, to our knowledge, never described. Irreversible loss of biologically important genetic information might also occur *in vivo* upon changing environments. Such presumably attenuated or non-pathogenic mutants might stay restricted, e.g., to the vector niche because they lost the ability to infect the host. They might even not survive in nature but only under artificial laboratory conditions where the metabolic burden of BadA expression would select for faster growing bacteria that silence the *badA* gene (e.g., via deletion or single bp mutations). However, this hypothesis has not been proven for *B. henselae* because suitable animal infection models mimicking human infections do not exist.

Overall, *badA* domain duplications, acquisitions, and deletions result in varying BadA proteins with different lengths and alternating repetitive domain arrangements, as is demonstrated within this rather small data set of eight *B. henselae* isolates. The diversity of BadA proteins illustrates the occurrence of active recombination events within the *badA* island, demonstrates the utilisation of the downstream *badA*-like domain region acting as a “toolbox” for rearrangements in *badA* composition, and strengthens our hypothesis of active phase variation via site-specific recombination in the scope of rapid host adaptation (e.g., pathogenicity and immune evasion).

## Conclusion

A detailed analysis of long-read sequenced genomes is required to discern subtle differences and numerous repeat sequences between closely related bacterial genotypes. *B. henselae* and BadA provide an excellent setting to analyse host adaptation and evolution of pathogenic proteins and might serve as an example for other TAAs. BadA is verified to be important for the initial host-pathogen interaction via binding to ECM proteins (e.g., Fn and Col-I). It is thus important to verify surface expression of BadA (or other TAAs of *Bartonella* spp.) before starting infection experiments. By studying the diversity of eight *B. henselae* strains (human and cat isolates), we have gathered evidence of active recombination within the repetitive *badA* genomic island and suggest that among the studied strains, *B. henselae* G-5436 is the evolutionary ancestor. The pathogenic life cycle of *B. henselae* might promote frequent recombination events leading to the combinatorial reshuffling of similar domain arrays and the emergence of variations among different BadA proteins that possibly contribute to host immune evasion and enhance long term and efficient survival in the differing host conditions.

## Data Availability Statement

The raw data supporting the conclusions of this article will be made available by the authors upon request, without undue reservation. The genome sequences of all sequenced *B. henselae* isolates, together with their corresponding SRA data, have been deposited in the NCBI GenBank database under BioProject PRJNA720375 with the following genome accession numbers: CP072904 (Marseille), CP072903 (ATCC49882T var-1), CP072902 (ATCC49882T var-2), CP072901 (Berlin-I), CP072900 (G-5436), CP072899 (88-64 Oklahoma), CP072898 (FR96/BK38), and CP072897 (FR96/BK3) (Supplementary Table 1). The genomic sequence of badA from *B. henselae* Marseille was deposited separately under the GenBank accession number MK993576.1.

## Author Contributions

VK, AT, and DL designed the study. AT and WB performed the experimental work. KH provided the electron microscopy. SC and JM performed the mass spectrometry. DV contributed to binding experiments. AS helped in cloning experiments and long-read sequencing. AT and VK wrote the manuscript. All authors approved the final manuscript.

## Conflict of Interest

The authors declare that the research was conducted in the absence of any commercial or financial relationships that could be construed as a potential conflict of interest.

## Publisher’s Note

All claims expressed in this article are solely those of the authors and do not necessarily represent those of their affiliated organizations, or those of the publisher, the editors and the reviewers. Any product that may be evaluated in this article, or claim that may be made by its manufacturer, is not guaranteed or endorsed by the publisher.

## Abbreviations

ANI: average nucleotide identity
BadA: *Bartonella* adhesin A
BALI: *Bartonella* liquid (medium)
BSA: bovine serum albumine
CBA: Columbia blood agar (plates)
CCS: circular consensus sequences (reads)
Col-I: collagen-I
DAPI: 4′,6-diamidino-2-phenylindole
DMF: dimethylformamide
ECM: extracellular matrix
Fn: fibronectin
FS: freeze substitution
HMW: high molecular weight
HPF: high-pressure freezing
MS: mass spectrometry
o/n: overnight
PLT: progressive lowering of temperature
RT: room temperature
SMRT: single-molecule real-time
TAA: trimeric autotransporter adhesin
TMB: 3,3′,5,5′-tetramethylbenzidine (liquid substrate).
