# Supplementary material for: Long-Read Sequencing Reveals Genetic Adaptation of Bartonella Adhesin A Among Different Bartonella henselae Isolates
Source: Front Microbiol. 2022 Feb 7;13:838267. doi: 10.3389/fmicb.2022.838267 (PMC8859334; doi:10.3389/fmicb.2022.838267)
Supplement: Supplementary file 1 [file Data_Sheet_1.PDF]

Supplementary Material

Supplementary Figure 1: Mass spectrometry analysis of purified BadA from *B. henselae* Marseille. For immunisation of rabbits, fibers from *B. henselae* Marseille were purified (see Material and Methods) and the HMW protein was identified as BadA using mass spectrometry. Grey highlighted stretches in the BadA protein sequence are mass spectrometry-identified peptides.

```
MKKLSVTSKRQYNLYASPISRRLSLLMKLSLETVTVMFLLGASPVLASNLALTGAKNLSQNSPGVNYSKGSHGSIVLSGD
DDFCGADYVLGRGGNSTVRNGIPISVEEEYERFVKQKLMNNATSPYSQSSEQQVWTGDGLTSKGSGYMGGKSTDGDKNIL
PEAYGIYSFATGCGSSAQGNYSVAFGANATALTGGSQAFGVAALASGRVSVAIGVGSEATGEAGVSLGGLSKAAGARSVA
IGTRAKAQGEESIAIGSSVKNGDKDGSAVAQGAKAIAIGSNSISFQHYAVAVGAKAHALLSKTVALGYDSVADVDAGIRG
YDPVEDEPSKDVSFVWKSSLGAVSVGNRKEGLTRQIIGVAAGTEDTDAVNVAQLKALRGMISEKGGWNLTVNNDNNTVVS
SGGALDLSSGSKNLKIVKDGKKNNVTFDVARDLTLKSIKLDGVTLNETGLFIANGPQITASGINAGSQKITGVAEGTDAN
DAVNFGQLKKIETEVKEQVAASGFVKQDSDTKYLTIGKDTDGDTINIANNKSDKRTLTGIKEGDISKDSSEAITGSQLFT
TNQNVKTVSDNLQTAATNIAKTFGGGAKYEDGEWIAPAFKVKTVTGEGKEEEKRYQNVADALAGVGSSITNVQNKVTEQV
NNAITKVEGDALLWSDEANAFVARHEKSKLGKGASKATQENSKITYLLDGDVSKDSTDAITGKQLYSLGDKIASYLGGNA
KYEDGEWTAPTFKVKTVKEDGKEEEKTYQNVAEALTGVGTSFTNVKNEITKQINHLQSDDSAVVHYDKNKDETGGINYAS
VTLGKGKDSAAVTLHNVADGSISKDSRDAINGSQIYSLNEQLATYFGGGAKYENGQWTAPIFKVKTVKEDGEEEEKTYQN
VAEALTGVGTSFTNIKSEITKQIANEISSVTGDSLVKKDLATNLITIGKEVAGTEINIASVSKADRTLSGVKEAVKDNEA
VNKGQLDKGLKHLSDSLQSDDSAVVHYDKKTDETGGINYTSVTLGGKDKTPVALHNVADGSISKDSHDAINGGQIHTIGE
DVAKFLGGAASFNNGAFTGPTYKLSNIDAKGDVQQSEFKDIGSAFAGLDTNIKNVNNNVTNKFNELTQNITNVTQQVKGD
ALLWSDEANAFVARHEKSKLGKGASKATQENSKITYLLDGDVSKDSTDAITGKQLYSLGDKIASYLGGNAKYENGEWTAP
TFKVKTVKEDGKEEEKTYQNVAEALTGVGASFTNVKNEITKQINHLQSDDSAVVHYDKNKDETGGINYASVTLGKGKDSA
AVTLHNVADGSISKDSRDAINGSQIYSLNEQLATYFGGGAKYENGQWTAPIFKVKTVKEDGEEEEKTYQNVAEALTGVGT
SFTNIKSEITKQIANEISSVTGDSLVKKDLATNLITIGKEVAGTEINIASVSKADRTLSGVKEAVKDNEAVNKGQLDKGL
KHLSDSLQSDDSAVVHYDKKTDETGGINYTSVTLGGKDKTPVALHNVADGSISKDSHDAINGGQIHTIGEDVAKFLGGAA
SFNNGAFTGPTYKLSNIDAKGDVQQSEFKDIGSAFAGLDTNIKNVNNNVTNKFNELTQNITNVTQQVKGDALLWSDEANA
FVARHEKSKLGKGASKATQENSKITYLLDGDVSKDSTDAITGKQLYSLGDKIASYLGGNAKYENGEWTAPTFKVKTVKED
GKEEEKTYQNVAEALTGVGASFTNVKNEITKQINHLQSDDSAVVHYDKNKDETGGINYASVTLGKGKDSAAVTLHNVADG
SISKDSRDAINGSQIYSLNEQLATYFGGGAKYENGQWTAPIFKVKTVKEDGEEEEKTYQNVAEALTGVGTSFTNIKSEIT
KQIANEISSVTGDSLVKKDLATNLITIGKEVAGTEINIASVSKADRTLSGVKEAVKDNEAVNKGQLDTNIKKVEDKLTEA
VGKVTQQVKGDALLWSNEDNAFVADHGKDSAKTKSKITHLLDGNIASGSTDAVTGGQLYSLNEQLATYFGGGAKYENGQW
TAPTFKVKTVNGEGKEEEQTYQNVAEALTGVGASFMNVQNKITNEITNQVNNAITKVEGDSLVKQDNLGIITLGKERGGL
KVDFANRDGLDRTLSGVKEAVNDNEAVNKGQLDADISKVNNNVTNKFNELTQNITNVTQQVKGDALLWSDEANAFVARHE
KSKLEKGVSKATQENSKITYLLDGDISKGSTDAVTGGQLYSLNEQLATYFGGGAKYENGQWTAPTFKVKTVNGEGKEEEQ
TYQNVAAAFEGVGTSFTNIKSEITKQINNEIINVKGDSLVKRDLATNLITIGKEIEGSVINIANKSGEARTISGVKEAVK
DNEAVNKGQLDTNIKKVEDKLTEAVGKVTQQVKGDALLWSNEDNAFVADHGKDSAKTKSKITHLLDGNIASGSTDAVTGG
QLYSLNEQLATYFGGGAKYENGQWTAPTFKVKTVNGEGKEEEKTYQNVAAAFEGVGTSFTNIKSEITKQIANEISNVTGD
SLVKKDLDTNLITIGKEIAGTEINIASVSKADRTLSGVKEAVNDNEAVNKGQLDANISKVNNNVTNKFNELTQSITNVTQ
QVKGDALLWSDEANAFVARHEKSKLEKGVSKATQENSKITYLLDGDISKGSTDAVTGGQLYSLNEQLATYFGGGAKYENG
QWTAPTFKVKTVNGEGKEEEQTYQNVAAAFEGVGTSFTNIKSEITKQINNEIINVKGDSLVKRDLATNLITIGKEIEGSV
INIANKSGEARTISGVKEAVKDNEAVNKGQLDTNIKKVEDKLTEAVGKVTQQVKGDALLWSNEDNAFVADHGKDSAKTKS
KITHLLDGNIASGSTDAVTGGQLYSLNEQLATYFGGGAKYENGQWTAPTFKVKTVNGEGKEEEKTYQNVAAAFEGVGTSF
TNIKSEITKQIANEISNVTGDSLVKKDLDTNLITIGKEIAGTEINIASVSKADRTLSGVKEAVNDNEAVNKGQLDANISK
VNNNVTNKFNELTQSITNVTQQVKGDALLWSDEANAFVARHEKSKLEKGVSKATQENSKITYLLDGDISKGSTDAVTGGQ
LYSLNEQLATYFGGGAKYENGQWTAPTFKVKTVNGEGKEEEQTYQNVAAAFEGVGTSFTNIKSEITKQINNEIINVKGDS
LVKRDLATNLITIGKEIEGSVINIANKSGEARTISGVKEAVKDNEAVNKGQLDTNIKKVEDKLTEAVGKVTQQVKGDALL
WSNEDNAFVADHGKDSAKTKSKITHLLDGNIASGSTDAVTGGQLYSLNEQLATYFGGGAKYENGQWTAPTFKVKTVNGEG
KEEEKTYQNVAAAFEGVGTSFTHVKNEITKQINHLQSDDSAVVHYDKDDKNGSINYASVTLGKGKDSAAVALHNVADGSI
SKDSHDAINGGQIHTIGEDVAKFLGGDAAFKDGAFTGPTYKLSNIDAKGDVQQSEFKDIGSAFAGLDTNIKNVNNNVTNK
LSELTQNITTVTQQVKGNALLWSDEANAFVARHEKSKLEKGASKAIQENSKITYLLDGDVSKGSTDAVTGGQLYSMSNML
ATYLGGNAKYENGEWTAPTFKVKTVNGEGKEEEQTYQNVAEALTGVGTSFTNIKSEIAKQINHLQSDDSAVIHYDKNKDE
TGTINYASVTLGKGEDSAAVALHNVAAGNIAKDSRDAINGSQLYSLNEQLLTYFGGDAGYKDGQWIAPKFHVLQFKSDGS
SGEKESYDNVAAAFEGVNKSLAGMNERINNVTAGQNVSSSSLNWNETEGGYDARHNGVDSKLTHVENGDVSEKSKEAVNG
SQLWNTNEKVEAVEKDVKNIEKKVQDIATVADSAVKYEKDSTGKKTNVIKLVGGSESEPVLIDNVADGKIEADSKQAVNG
GQLRDYTEKQMKIVLDDAKKYTDERFNDVVNNGINEAKAYTDVKFEALSYTVEEVRKEARQAAAIGLAVSNLRYYDIPGS
LSLSFGTGIWRSQSAFAIGAGYTSEDGNIRSNLSITSSGGQWGVGAGITLRLK
```

Supplementary Table 1: Genome size, sequencing data, and GenBank accession number for eight sequenced *B. henselae* genomes (GC-content is 38% for all strains).

B. henselae **strain**	**Single contig genome size (bp)**	**Mean coverage**[a]	**N50 subread length (bp)**[b]	**Subreads**	**Mean subread length (bp)**	**CCS reads (≥Q20)**[c]	**CCS mean read length (≥Q20**[b]**, bp)**	**Predicted CDSs**	**GenBank accession number**
Marseille	1,906,759	802x	9,778	335,562	7,486	18,542	7,486	1,566	CP072904
ATCC49882^T var-1	1,955,459	1,113x	9,778	631,218	5,801	28,343	5,801	1,574	CP072903
ATCC49882^T var-2	1,931,585	651x	10,446	306,776	8,307	10,695	6,051	1,579	CP072902
Berlin-I	1,931,655	652x	10,446	238,737	9,695	9,490	7,440	1,584	CP072901
G-5436	1,963,796	629x	10,446	255,359	9,561	9,597	7,025	1,577	CP072900
88-64 Oklahoma	1,969,298	566x	10,446	215,349	9,663	8,300	7,157	1,610	CP072899
FR96/BK38	1,944,393	762x	10,446	371,120	8,161	12,787	8,792	1,561	CP072898
FR96/BK3	1,935,288	922x	10,446	356,346	9,460	13,834	7,089	1,571	CP072897

[a] Total #bases/genome size

[b] for entire SMRT cell

[c] no Q-filtered reads for Marseille and ATCC49882^T var-1

Supplementary Table 2: Primers used in this study [a].

Primer	5’-3’ sequence
FrUp_Fw	AGCCCGGGGGATCCATTGGTTTGGCAGTGTCCAACT
FrUp_Rv	CCTGCGCCTCTCTTTGATGTGACAGATAATTTTTTCATAATAAATTCTCCAA
FrDown_Fw	AAAGAGAGGCGCAGGGATTACTTTGAG
FrDown_Rv	GGCCGCTCTAGAACTAGACTTCAAATAATATCTCTTACTATTGAATAATATTTTCCTAAAACTACG
pBIISK_Fw	CTAGTTCTAGAGCGGCCGC
pBIISK_Rv	TGGATCCCCCGGGCTG
IntegrationA_Fw	GGATCCATTGGTTTGGCAGTG
IntegrationA_Rv	GTTTTTCCATGGTAGCATCACTC
IntegrationB_Fw	TTTAAGCTACGCGGTTGAGGATG
IntegrationB_Rv	CGCTCTAGAACTAGACTTCAAATAATATCTCTTAC
Repeat_Fw	GAGATTGCTAATGCGAATGG
Repeat_Rv	ATTACCAACACCATTGCC

[a] Underlined regions create overhangs used for Gibson cloning

Supplementary Table 3: Location of the ORFs in the *badA* island (see also Fig. 2).

	Marseille	ATCC49882^T var-1	ATCC49882^T var-2	Berlin-I	G-5436	88-64 Oklahoma	FR96/BK38	FR96/BK3
***badA* pseudogene**								
NCBI locus tag	KAE73_05700	KAE76_05665	KAE74_05750	KAE72_05775	KAE71_04240	KAE77_04380	KAE75_04135	KAE70_05705
Start position	1,353,514	1,383,089	1,383,227	1,383,299	945,048	949,628	923,247	1,379,217
Size (bp)	5,361	5,181	5,181	5,181	5,181	5,181	5,430	5,430
badA								
NCBI locus tag	KAE73_05715 and MK993576	*bapA* and KAE76_05685	KAE74_05770… KAE74_05780	KAE72_05790… KAE72_05800	KAE71_04225	KAE77_04365	KAE75_04120	KAE70_05720
Start position	1,362,098	1,391,491 and 1,396,823 [a]	1,391,629	1,391,701	941,826	935,475	909,103	1,387,870
Size (bp)	11,922	5,331 and 5,331	8,763	8,762	10,926	10,932	10,926	13,224
***badA*-like domain region**								
NCBI locus tag	/	KAE76_05690… KAE76_05770	/	/	KAE71_04220…KAE71_04150	/	KAE75_04115…KAE75_04045	/
Start position	/	1,402,734 [a]	/	/	930,320 [a]	/	908,530 [a]	/
Size (bp)	/	21,532	/	/	21,532	/	20,510	/

[a] NCBI annotations and ORFs in Figure 2 differ slightly from each other due to manual adjustment of some ORFs.
